# EGFR T790M mutation as a novel target for immunotherapy against EGFR-TKI-resistant non-small cell lung cancer

**DOI:** 10.1186/2051-1426-1-S1-P235

**Published:** 2013-11-07

**Authors:** Tetsuro Sasada, Teppei Yamada, Koichi Azuma, Satoko Matsueda, Kyogo Itoh

**Affiliations:** 1 Department of Immunology and Immunotherapy, Kurume University School of Medicine, Kurume, Japan; 2 Cancer Vaccine Center, Kurume University, Kurume, Japan; 3 Department of Internal Medicine, Kurume University School of Medicine, Kurume, Japan

## 

Epidermal growth factor receptor tyrosine kinase inhibitors (EGFR-TKIs), such as gefitinib and erlotinib, have achieved high clinical response rates in patients with non-small cell lung cancers (NSCLCs), which possess somatic mutations in the tyrosine kinase domain of the epidermal growth factor receptor (EGFR) gene. However, over time (median of 6-12 months), most tumors are known to develop acquired resistance to EGFR TKIs. Currently, there have been no effective treatment options against NSCLC patients with the secondary T790M resistance mutation, which occurs in 50% of patients with acquired resistance to EGFR-TKIs. Here we identified two novel HLA-A2-restricted T cell epitopes derived from the T790M resistance mutation of EGFR, T790M-5 (MQLMPFGCLL) and T790M-7 (LIMQLMPFGCL), as a potential target for immunotherapy against EGFR-TKI-resistant patients. When peripheral blood cells were repeatedly stimulated in vitro with these two peptides and assessed by antigen-specific IFN-γ secretion, T cell lines specific to T790M-5 and T790M-7 could be established in 5 of 6 (83%) and 3 of 6 (50%) healthy donors, respectively. Additionally, the T790M-5- and T790M-7-specific CTLs displayed an MHC class I-restricted reactivity against NSCLC cell lines expressing both HLA-A2 and the T790M mutation. More interestingly, antigen-specific T cell responses to these epitopes were more frequently observed in EGFR-TKI-sensitive patients (3 of 6, 50%) than in EGFR-TKI-resistant patients (2 of 11, 18%), suggesting the possibility that immune responses to the T790M-derived neo-antigens might prevent acquisition of EGFR-TKI resistance in NSCLC patients by eliminating cancer cells with the T790M mutation. Given high immunogenicity in human T cells, these epitopes could provide a novel immunotherapeutic approach against NSCLC patients with the T790M mutation. Since T cell repertoires with high avidities to the “neo-antigens” derived from mutated amino acid sequences are expected to be available due to lack of central and/or peripheral T cell tolerance, the identified T790M-derived T cell epitopes might be a highly attractive target for immunotherapeutic manipulation.

